# Spatial frequency channels depend on stimulus bandwidth in normal and amblyopic vision: an exploratory factor analysis

**DOI:** 10.3389/fncom.2023.1241455

**Published:** 2023-10-24

**Authors:** Alexandre Reynaud, Seung Hyun Min

**Affiliations:** ^1^McGill Vision Research, Department of Ophthalmology and Visual Sciences, McGill University, Montréal, QC, Canada; ^2^Research Institute of the McGill University Health Center, Montréal, QC, Canada; ^3^State Key Laboratory of Ophthalmology, Optometry and Vision Science, School of Ophthalmology and Optometry, Affiliated Eye Hospital, Wenzhou Medical University, Wenzhou, Zhejiang, China

**Keywords:** contrast sensitivity, amblyopia, factor analysis, spatial frequency channels, noise

## Abstract

The Contrast Sensitivity Function (CSF) is the measure of an observer’s contrast sensitivity as a function of spatial frequency. It is a sensitive measure to assess visual function in fundamental and clinical settings. Human contrast sensitivity is subserved by different spatial frequency channels. Also, it is known that amblyopes have deficits in contrast sensitivity, particularly at high spatial frequencies. Therefore, the aim of this study was to assess whether the contrast sensitivity function is subtended by the same spatial frequency channels in control and amblyopic populations. To determine these spatial frequency channels, we performed an exploratory factor analysis on five datasets of contrasts sensitivity functions of amblyopic and control participants measured using either gratings or noise patches, taken from our previous studies. In the range of 0.25–10 c/d, we identified two spatial frequency channels. When the CSF was measured with noise patches, the spatial frequency channels presented very similar tuning in the amblyopic eye and the fellow eye and were also similar to what was observed in controls. The only major difference was that the weight attributed to the high frequency channel was reduced by approximately 50% in the amblyopic eye. However, when the CSF was measured using gratings, the spatial frequency channels of the amblyopic eye were tuned toward lower spatial frequencies. These findings suggest that there is no mechanistic deficit for contrast sensitivity in amblyopia and that amblyopic vision may just be subjected to excessive internal noise and attenuation at higher spatial frequencies, thereby supporting the use of therapeutic strategies that involve rebalancing contrast.

## Introduction

Spatial frequency channels within the human visual system enable observers to detect modulations of luminance (De Valois and De Valois, 1991; Peterzell and Teller, 1996), color (Peterzell and Teller, 2000), disparity (Serrano-Pedraza and Read, 2010; Reynaud and Hess, 2017), or second-order patterns (Landy and Oruç, 2002; Ellemberg et al., 2006; Reynaud and Hess, 2012). In the case for perceiving luminance modulation, two channels have been known to subserve a spatial frequency range from 0.1 to 10 c/d: one is tuned for low spatial frequency with a peak around 1 c/d and another for high spatial frequency with a peak around 2 c/d (Peterzell and Teller, 1996).

Individuals with amblyopia exhibit deficits in contrast sensitivity within their amblyopic eye, notably at high spatial frequencies (Hess and Howell, 1977; Gao et al., 2015). Therefore, whether the channel for high spatial frequency in amblyopia is intact or impaired is an important question. To address this issue, we investigated whether the spatial frequency channels subtend contrast sensitivity in amblyopia similarly to those in normal observers. Are they the same as those from normal observers or is there a specific deficit in the high frequency channel? Frequency channels can be assessed using various psychophysical protocols such as contrast adaptation (Blakemore et al., 1970, 1973), identification at threshold (Watson and Robson, 1981; Ellemberg et al., 2006; Reynaud and Hess, 2012), masking (Stromeyer et al., 1982; Greenlee and Magnussen, 1988), or using statistical methods such as factor analysis (Sekuler et al., 1984; Billock and Harding, 1996; Peterzell and Teller, 1996; Simpson and McFadden, 2005).

The goal of exploratory factor analysis is to reduce the number of dimensions from a high-dimensional dataset. In this paper, we will use the term *exploratory factor analysis* to refer to both principal component analysis and the common factor analysis methods despite their differences (Larsen and Warne, 2010). For this reason, we will interchangeably use the terms *factors* and *components* to denote spatial channels that underlie the contrast sensitivity data across multiple spatial frequencies. By employing the multivariate method, we attempted to find the minimum number of dimensions that could capture the majority of variance from the data. Factor analysis of contrast sensitivity data is often begun by examining whether there are robust local correlations or covariances in the contrast sensitivity data of individuals between adjacent spatial frequencies to eventually determine common mechanisms. It requires a large number of measurements and sample size. To obtain such a large dataset, we re-analyzed the data of five of our previously published articles (Reynaud et al., 2014; Gao et al., 2015; Kim et al., 2017; Zhou et al., 2018; Beylerian et al., 2020). This enabled us to capture a wide range of spatial frequency where normal and amblyopic visual systems process information.

Amblyopes can perceive spatially distorted images within their amblyopic eye (Hess et al., 1978; Bedell et al., 1985) due to a strong internal noise in their visual system (Levi and Klein, 2003; Xu et al., 2006; Levi et al., 2007). One way to evaluate internal noise is by comparing it to external noise, i.e., input noise or stimulus noise (Baldwin et al., 2016). So, in the second part of this study, we examined in detail how stimulus noise could perturb the spatial channels that subserve different ranges of spatial frequency. To do so, we compared the tuning of the spatial channels measured with two distinct types of visual stimuli containing various amounts of noise in terms of orientation and spatial frequency bandwidth: broadband noise patterns and narrowband Gabor patches.

## Methods

We applied exploratory factor analysis to discover whether there are common sources of variance across unique variables from a large dataset. For instance, if the correlations across the variables are not independent, then the correlations can vary in a similar fashion within a group of variables but not in others. If the variance across different explanatory variables is independent from one another, then there is no underlying factor that controls the variance of these variables at the same time. However, if the variables display dependence, then we can deduce that there are common sources (or factors) of variance. In the case of contrast sensitivity data, it is widely known that there are common sources of variance across different spatial frequency levels. For this reason, we conducted factor analysis using five datasets from previous studies: 51 controls from Kim et al. (2017); 52 controls from Reynaud et al. (2014); 15 amblyopes and 17 controls from Beylerian et al. (2020); 8 amblyopes and 10 controls from Zhou et al. (2018); and 15 amblyopes out of 28 from Gao et al. (2015) (this dataset was partially corrupted) for a grand total of 130 controls and 38 amblyopes (Table 1). The new compiled dataset is openly available to download from the authors’ website: https://mvr.mcgill.ca/AlexR/data_en.html.

**Table 1:** Stimulus parameters and observers used in the analyzed studies.

Study	Stimulus characteristics			
	Size	Duration	Frequency range	Participants
Reynaud et al. (2014)	10°	1,000 ms	1–14.16 c/d	52 controls
Zhou et al. (2018)	3°	1,000 ms	0.25–9.57c/d	8 amblyopes and 10 controls
Beylerian et al. (2020)	3°	500 ms	0.25–6.87c/d	15 amblyopes and 17 controls
Kim et al. (2017)	5°	1,000 ms	0.24–9.57 c/d	51 controls
Gao et al. (2015)	10°	1,000 ms	1–14.16 c/d	15 amblyopes

In all papers, subjects’ contrast sensitivity functions were measured using the qCSF method (Hou et al., 2010; Lesmes et al., 2010) and a one-interval identification paradigm where the subject’s task was to identify the orientation of a noise pattern. Additionally, in the second part of the paper, we analyzed the contrast sensitivity measured using Gabor patches from Zhou et al. (2018) and Beylerian et al. (2020). To gather a sufficient number of observations for factor analysis, and as experimental conditions were almost identical (see Table 1) the datasets from Zhou et al. (2018) and Beylerian et al. (2020) were merged and truncated to the frequency range 0.25 to 6.87 c/d. All data analysis was performed using Matlab 2019b (the Mathworks).

## Results

**Figure 1:**
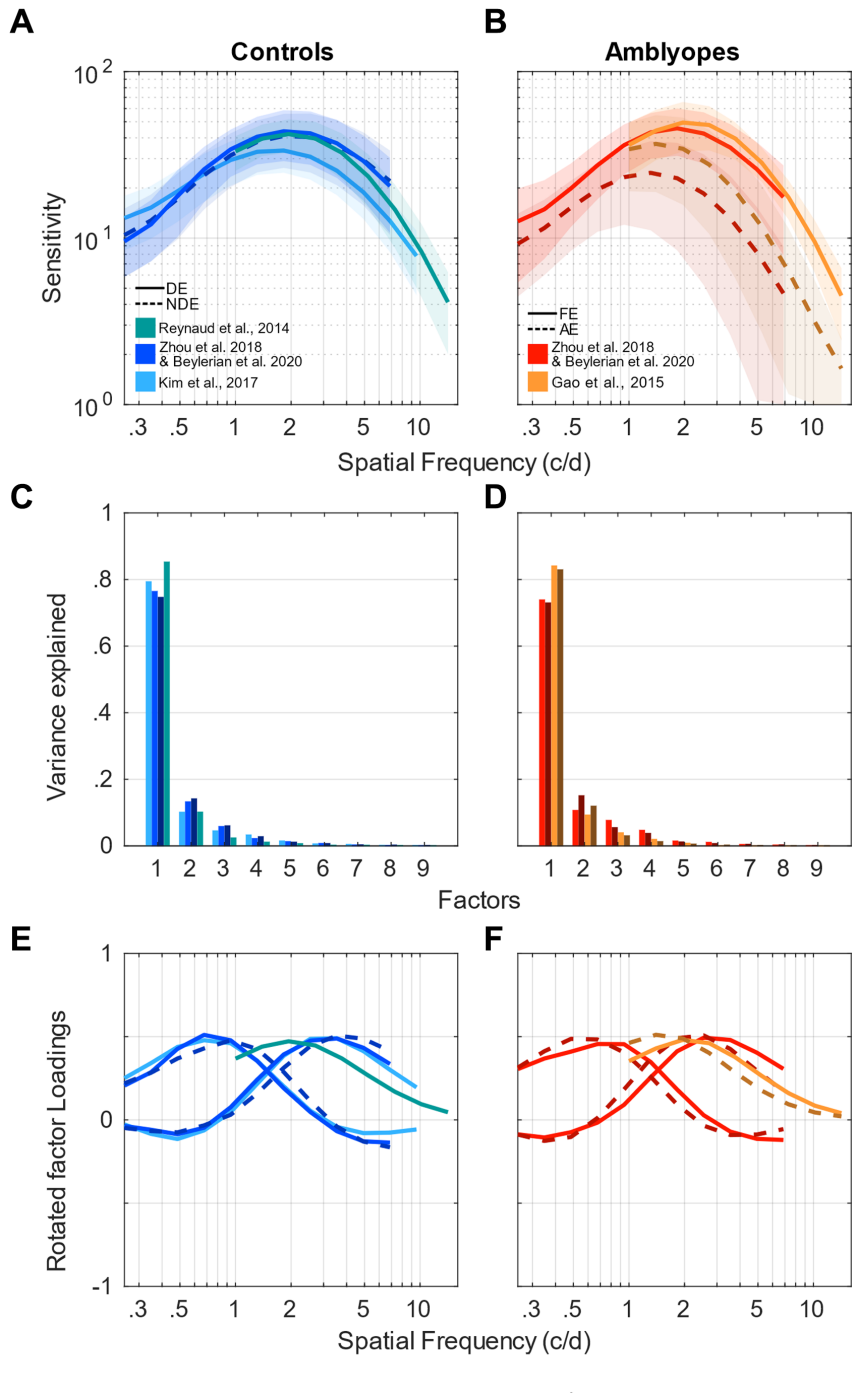
Factor analysis of contrast sensitivity functions. **(A)** Contrast sensitivity functions of control participants taken from Kim et al. (2017) (light blue); Reynaud et al. (2014) (green); Beylerian et al. (2020) and Zhou et al., 2018 (medium blue). Solid clear lines represent the dominant eye and darker dashed lines represent the non-dominant eye. Shaded areas represent ± std. **(B)** Contrast sensitivity functions of amblyopic participants taken from Gao et al. (2015) (orange); Beylerian et al. (2020) and Zhou et al. (2018) (red). Solid clear lines represent the fellow eye and darker dashed lines represent the amblyopic eye. Shaded areas represent ± std. **(C)** Scree plot presenting the explained variance from the singular value decomposition (SVD) for the 9 first factors of the control participants’ sensitivity. Same colors as in **(A)**. **(D)** Scree plot presenting the explained variance from the singular value decomposition (SVD) for the 9 first factors of the amblyopic participants’ sensitivity. Same colors as in **(B)**. **(E)** First factors following a varimax rotation for the control participants’ sensitivity. One or 2 factors were chosen for each study, indicated by different colors and linestyles as in **(A)**. **(F)** First factors following a varimax rotation for the amblyopic participants’ sensitivity. One or 2 factors were chosen for each study, indicated by different colors and line styles as in **(B)**.

The averaged contrast sensitivity functions (CSF) measured using noise patterns from the analyzed studies are presented in Figures 1A,B for control and amblyopic subjects, respectively. Although the protocols, equipment and the range of tested spatial frequencies were slightly different across studies, the measured CSFs are remarkably similar (see Figures 1A,B). For both eyes of controls and the fellow eye of amblyopes, they peak around 2 c/d at a sensitivity of approximately 50. The sensitivity of the amblyopic eye is reduced and peaks at a lower frequency, around 1 c/d (dashed curves in Figure 1B). The only noticeable difference between datasets is the amplitude of the CSF of the amblyopic eyes of amblyopes. It is approximately 40 in the data of Gao et al. (2015) (dark-orange dashed curve) and 25 in datasets of Zhou et al. (2018) and Beylerian et al. (2020) (dark-red dashed curve). However, their peaks are both at 1.5 c/d. This discrepancy is probably due to the variability within the amblyopic eye’s contrast sensitivity at the population level, thereby providing a strong motivation for us to use multivariate analysis methods such as factor analysis.

To determine the precise tuning of the spatial frequency channels across these studies, we performed an exploratory factor analysis on our contrast sensitivity datasets (Peterzell, 2016). In our case, the total number of dimensions (i.e., factors or components) from the datasets represents the number of tested spatial frequencies (see Table 1).

We conducted a singular value decomposition (SVD) to calculate the eigenvalues, which capture the proportion of variance explained by each factor. The eigenvalues for the 9 first factors of each dataset are represented in form of scree plots in Figure 1C for control subjects and Figure 1D for amblyopic subjects. The higher the eigenvalue, the higher the proportion of the variance by a specific factor or component. Table 2 shows the total accounted variance by 1 or 2 components for each dataset, as well as the number of components suggested by standard criterions: total variance explained >80%, Kaiser-Guttman criterion, broken-stick method, and random average under permutation (see a description of those methods in Peres-Neto et al., 2005). Despite some disagreements among the methods, they collectively indicated that 1–3 components should be kept depending on the dataset. Looking at the elbow and proportion of variance explained in those scree plots, we decided to use a criterion of 80% of variance explained to determine the number of components to use in our analysis (O’Rourke and Hatcher, 2013). Where the scree “breaks” in the plot represents the demarcation between factors that account for the data’s variance significantly and those that do not. Based on these criteria, we selected 2 components in studies where a relatively low spatial frequency range was tested and only 1 component in studies where a higher spatial frequency range was tested (see Table 2).

**Table 2:** Variance explained by the first or the two-first components in each dataset.

Study	#Dimensions	DE/FE						NDE/AE					
		Explained variance		#Suggested components				Explained variance		#Suggested components			
		Factor 1	Factor 1+2	80%	KG	BS	RP	Factor 1	Factor 1+2	80%	KG	BS	RP
Reynaud et al. (2014)	9	0.85	0.95	1	2	1	2						
Zhou et al. (2018) and Beylerian et al. (2020) (controls)	11	0.76	0.90	2	2	1	2	0.75	0.89	2	2	1	2
Zhou et al. (2018) and Beylerian et al. (2020) (amblyopes)	11	0.74	0.84	2	3	1	3	0.73	0.88	2	2	1	2
Kim et al. (2017)	12	0.79	0.89	2	2	1	3						
Gao et al. (2015)	9	0.84	0.93	1	2	1	2	0.83	0.95	1	2	1	2

Number of components suggested by standard criterions: total variance explained >80% (column 80%), Kaiser-Guttman criterion (column KG), broken-stick method (column BS), and random average under permutation (column RP). For our exploratory factor analysis, we set the criterion for selecting the number of factors that could explain the total variance by 80%. Note that the range of tested spatial frequencies differ across studies (see Table 1).

Next, the factors were rotated using a varimax orthogonal rotation so that we could obtain a simple structure accounting for the channel tuning curves (Kaiser, 1958; Peterzell and Teller, 2000; Simpson and McFadden, 2005; Peterzell, 2016; Reynaud and Hess, 2017). The varimax rotation simplifies the interpretability of loadings and enhances identifiability of each factor. The tuning curves of the factors from controls are shown in Figure 1E and those from amblyopes in Figure 1F in the form of loadings. If the data from a certain range of spatial frequencies are highly correlated, then their loadings will be similar. For instance, loadings from the first component are large in the range of low spatial frequencies (about 0.7 c/d) but not at higher spatial frequencies (see Figure 1E). This indicates that the first component underlies the correlation of data at neighboring low spatial frequencies. Conversely, loadings from the second component peak at around 2 c/d. This indicates that the second component strongly accounts for the variance in the range of high spatial frequency (but not low spatial frequency). According to Figure 1B, it is evident that the amblyopic eye of the observers demonstrates a worse sensitivity and a peak at a lower spatial frequency than those of their fellow eye. However, based on their loadings (Figure 1F), it seems that the tunings of the spatial channels are similar between the two eyes. Specifically, loadings of the first and second components also, respectively, peak at about 0.7 c/d and 2 c/d for both eyes. In addition, they are similar to those of normal observers who have a much higher sensitivity and a peak at a higher spatial frequency (Figure 1E). In sum, these indicate that despite their differences in the sensitivity, the spatial channels are mechanistically intact in amblyopia.

Our first analysis revealed that the spatial channels in amblyopia themselves are unscathed. However, whether the direct contribution of these channels to spatial vision parallels to that of those in normal observer remains to be investigated. For this reason, we conducted an additional analysis, where we calculated the weights (i.e., contribution to the observed contrast sensitivity data) of these channels from the data of Zhou et al. (2018) and Beylerian et al. (2020).

**Figure 2:**
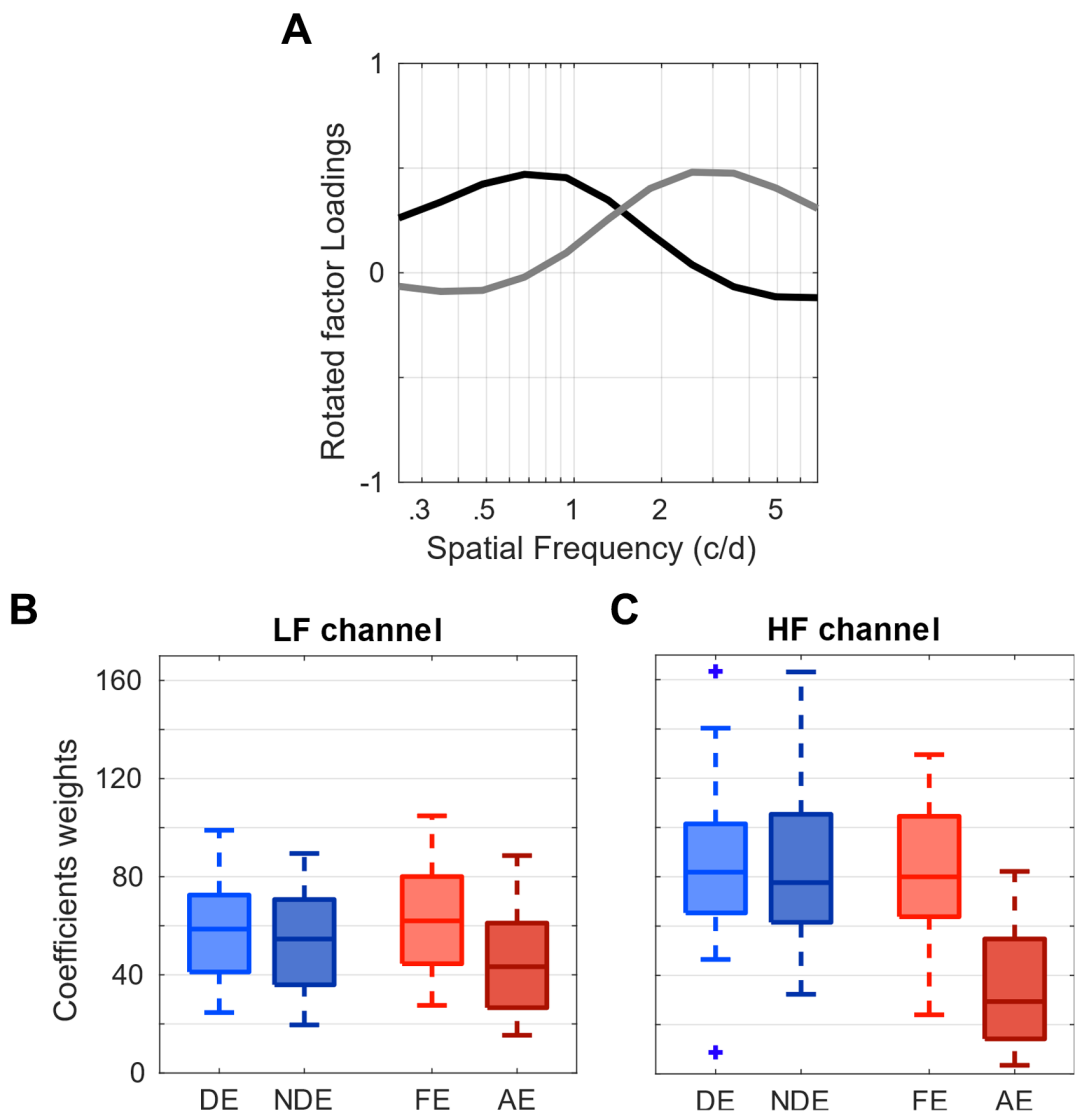
Contribution of spatial frequency channels to contrast sensitivity. **(A)** tuning curves of the low (black) and high (gray) spatial frequency channels determined by the factor analysis. **(B)** Distribution of weights of the low spatial-frequency channel from the 4 eye viewing conditions. **(C)** Distribution of weights of the high spatial-frequency channel from the 4 eye viewing conditions.

First, we averaged the loadings across four conditions, which correspond to each eye’s testing (DE, NDE, FE, and AE; Figure 2A). Subsequently, with the averaged loadings and the observed sensitivity data, we were able to compute the weights. To illustrate, the weights β of each of these channels were calculated for each individual sensitivity using the Moore-Penrose pseudo inverse X+ (Equation 1):


(1) $$\beta =X^{+}y$$

where *y* is the matrix of all individual sensitivities, *X*^+^ is the Moore-Penrose pseudo inverse of the new basis matrix *X* whose two columns represent the two factors and *β* is a two-rows matrix in which each column contains the pair of coefficients associated to the two factors estimated for each subject (Friston et al., 1995; Woolrich et al., 2004; Reynaud et al., 2011).

These pairs of coefficients correspond to the respective weights or contribution of each spatial channel (i.e., factor) to the observed contrast sensitivity data of each observer. The average and dispersion of the weights of the low and high frequency channels obtained for all eye conditions are represented as boxplots in Figures 2B,C, respectively. There is a significant difference in the distribution of weights in the low frequency channel (one-way ANOVA *p* < 0.01; Figure 2B) and an even more marked one in the high frequency channel, where the amblyopic eyes (AE) present strongly reduced weights (one-way ANOVA *p* < 0.001; Figure 2C). These findings demonstrate that the contribution of the high-spatial frequency channel from the amblyopic eye to contrast sensitivity is markedly less than that of the fellow eye.

**Figure 3:**
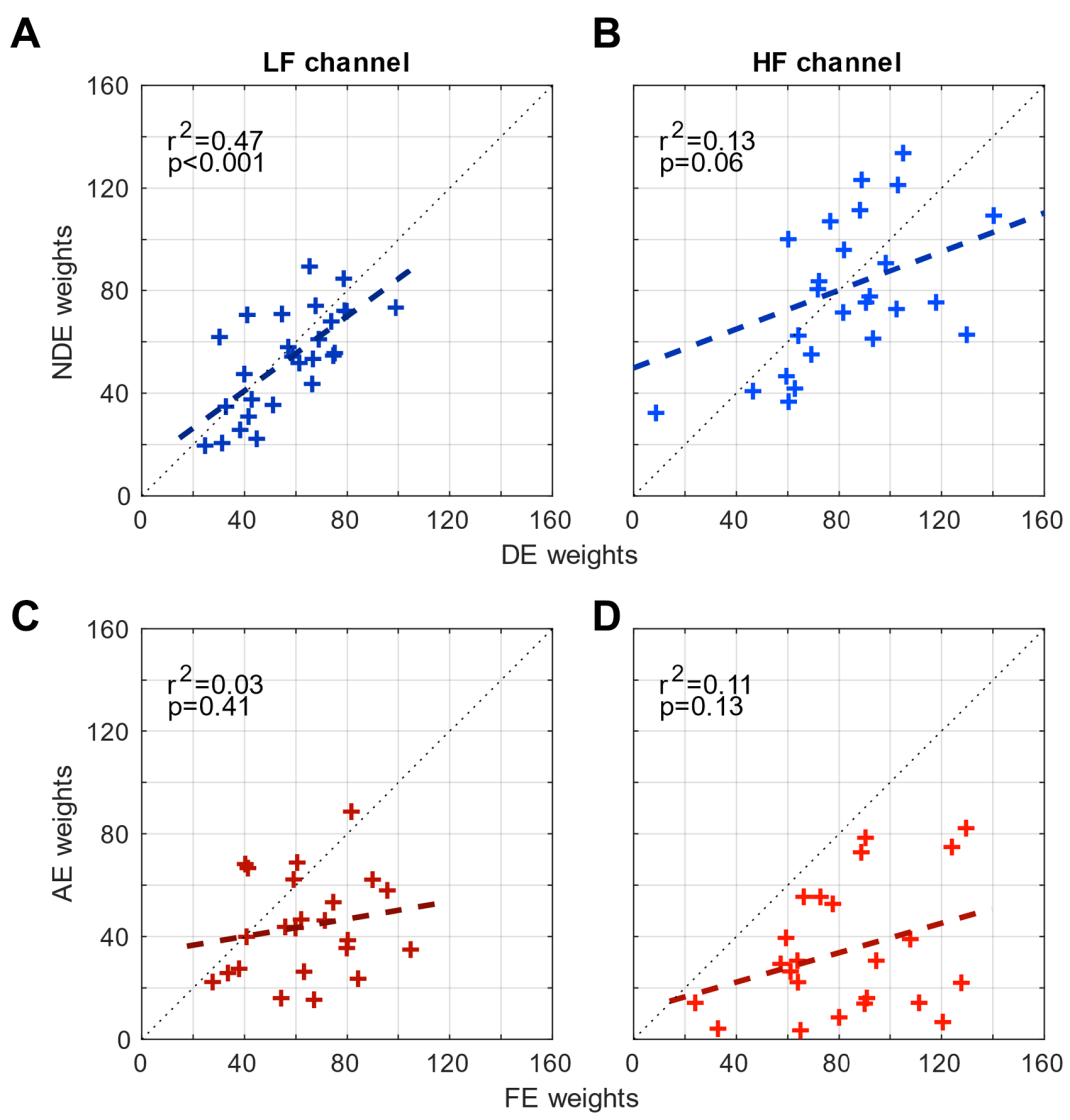
Comparison of the weights of the eyes. **(A)** Correlation between the weights of the NDE and the weights of the DE for controls in the low-frequency channel. **(B)** Correlation between the weights of the NDE and the weights of the DE in the high-frequency channel. **(C)** Correlation between the weights of the AE and the weights of the FE for amblyopes in the low-frequency channel. **(B)** Correlation between the weights of the AE and the weights of the FE in the high-frequency channel.

Thus far, we have shown that although the tunings of the spatial channels in amblyopia are mechanistically intact, the high-spatial frequency channel of the amblyopic eye contributes significantly less to contrast sensitivity than that of the fellow eye, indicating that there is a deficit in the spatial channel of the amblyopic eye. This illustrates the asymmetrical level of the contribution between the eyes to spatial vision in amblyopia. One way to quantify this asymmetry is to compute the correlation between the weights from both eyes in each range of spatial frequency (Figure 3). For controls, the weights of the 2 eyes are strongly correlated in the low-frequency channel (*r*^2^ = 0.47, *p* < 0.001; Figure 3A) but only mildly so in the high-frequency channel (*r*^2^ = 0.13, *p* = 0.06; Figure 3B). The presence of the strong correlation in controls shows that there is a symmetrical level of the contribution from the low spatial frequency channel to contrast sensitivity. However for amblyopes, the weights of the 2 eyes are not correlated in either of the two channels (*p* > 0.1; Figures 3C,D). This result quantitatively demonstrates that there is an asymmetry or mismatch in the contributions from the two eyes’ spatial channels at both low and high spatial frequencies in amblyopia.

**Figure 4:**
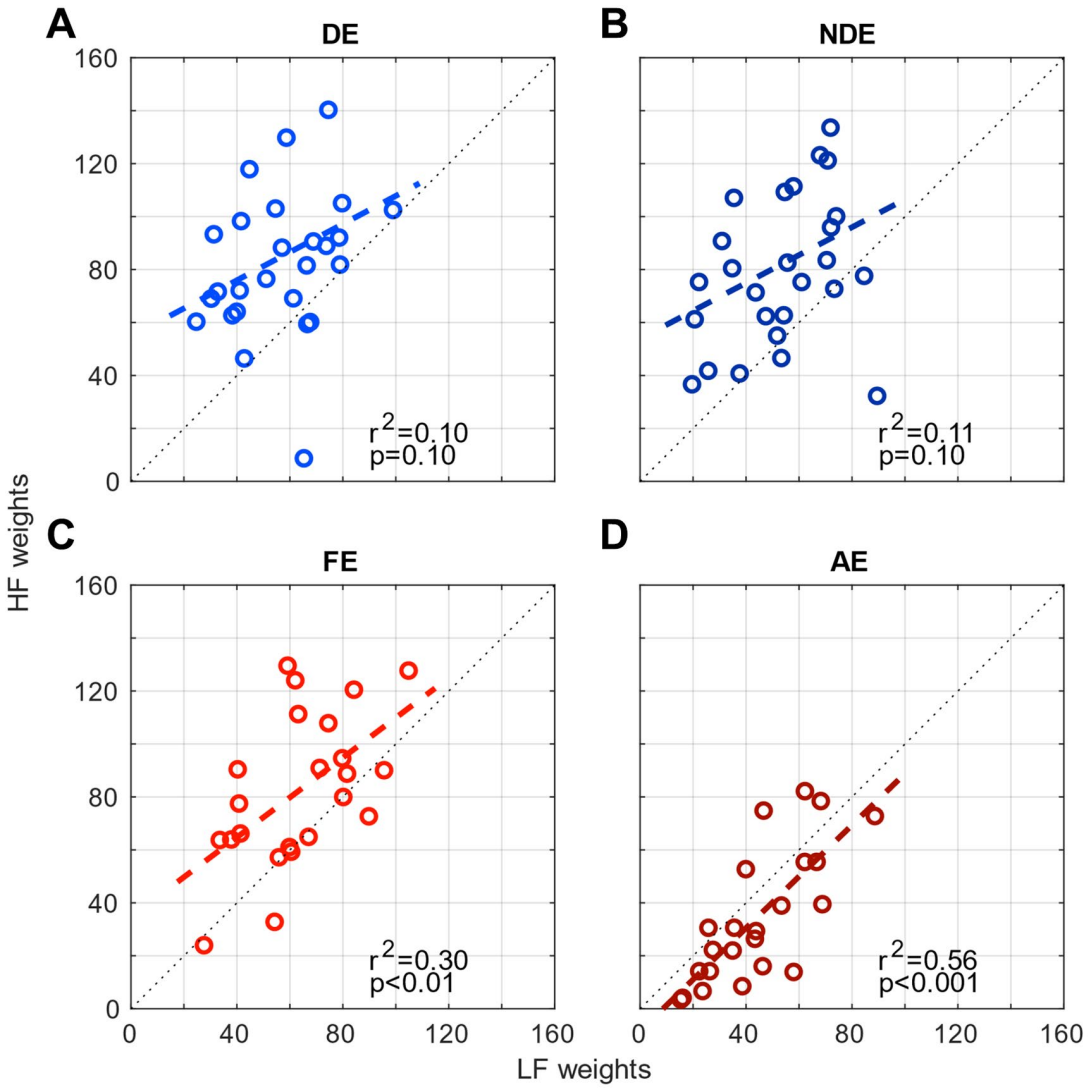
Comparison of the weights of the 2 channels (high-frequency HF and low-frequency LF) for each eye. **(A)** the dominant eye of controls, **(B)** the non-dominant eye of controls, **(C)** the fellow eye of amblyopes, and **(D)** the amblyopic eye of amblyopes.

Next, in light of the correlation results that demonstrated the different contribution levels between the eyes for both channels in amblyopia, we examined whether there would be a relationship between the contributions from the two spatial channels (weights from low- and high-spatial frequency ranges) within each eye. In other words, we wanted to investigate whether there would be a correlation between the weights from low spatial channel and that from the high spatial channel for each eye in both normal and amblyopic observers. The results are shown in Figure 4. We observed significant correlations (*p* < 0.05) in the amblyopic group FE (Figure 4C) and AE (Figure 4D) but only a mild correlation in the control group DE (*r*^2^ = 0.10, *p* = 0.10; Figure 4A) and NDE (*r*^2^ = 0.11, *p* = 0.10; Figure 4B). These findings indicate that, if a spatial channel from a range of spatial frequency within an eye exerts a strong contribution to contrast sensitivity, then the other channel also does so. This demonstrates that the strength of the weights is determined by the eye, rather than a specific range of spatial frequency where the channels are tuned to operate.

**Figure 5:**
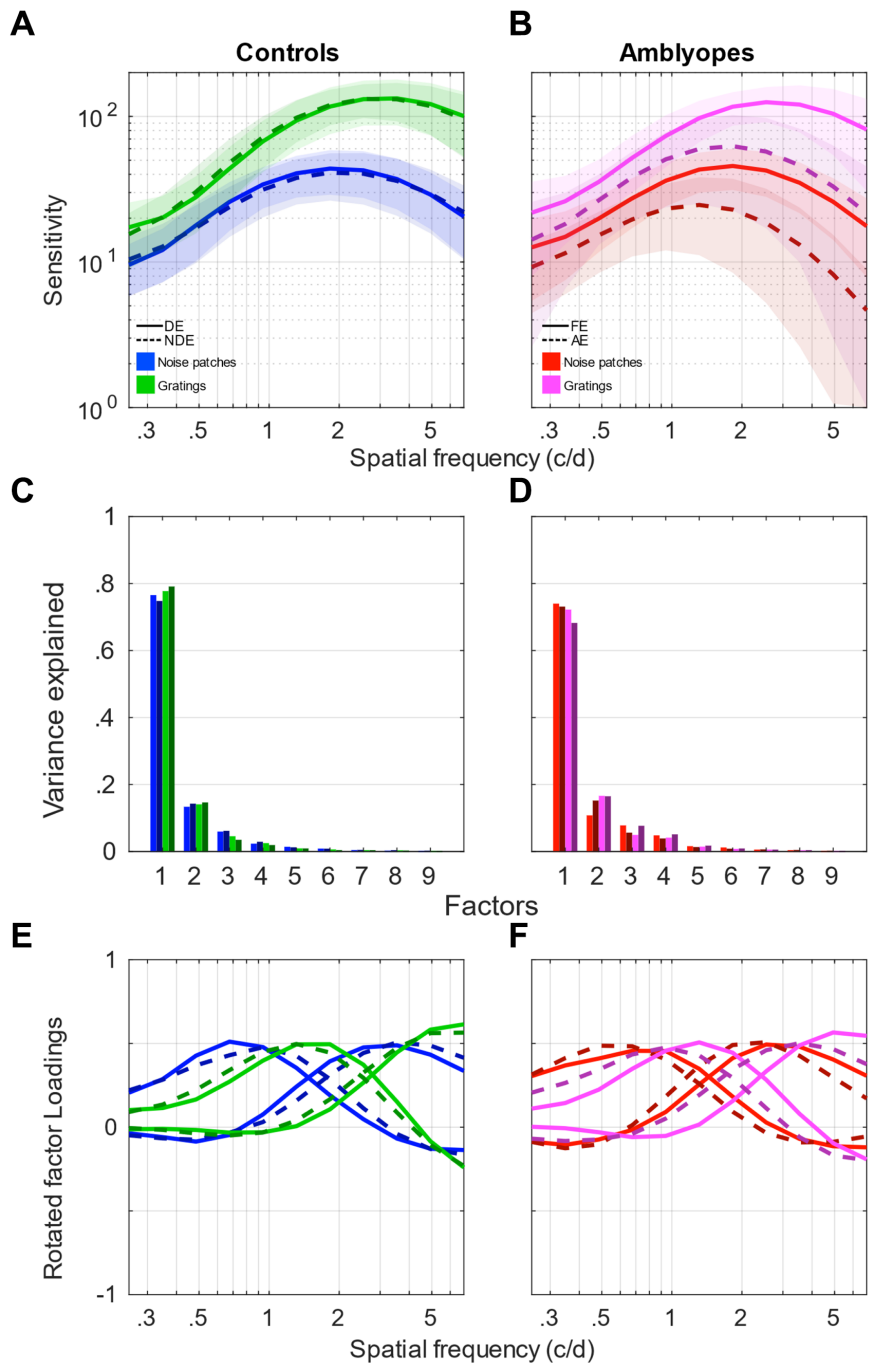
Factor analysis of contrast sensitivity functions compared between gratings and noise patches. **(A)** Contrast sensitivity functions of control participants measured using gratings (green) and noise patches (blue). Solid clear lines represent the dominant eye and darker dashed lines represent the non-dominant eye. Shaded areas represent ± std. **(B)** Contrast sensitivity functions of amblyopic participants measured using gratings (magenta) or noise patches (red). Solid clear lines represent the fellow eye and darker dashed lines represent the amblyopic eye. Shaded areas represent ± std. **(C)** Scree plot presenting the explained variance from the singular value decomposition (SVD) for the 9 first factors of the control participants’ sensitivity. Same colors as in **(A)**. **(D)** Scree plot presenting the explained variance from the singular value decomposition (SVD) for the 9 first factors of the amblyopic participants’ sensitivity. Same colors as in **(B)**. **(E)** First two factors following a varimax rotation for the control participants’ sensitivity. Same line style as in **(A)**. **(F)** First two factors following a varimax rotation for the amblyopic participants’ sensitivity. Same line style as in **(B)**. Data from Zhou et al. (2018) and Beylerian et al. (2020).

This first analysis showed that, surprisingly, controls and amblyopes present the same spatial frequency channels. However, the contribution to the high spatial frequency channel is reduced in amblyopia. So, in a second time, we wanted to investigate if those spatial frequency channels are affected by the orientation and spatial frequency bandwidth of the stimulus. We repeated the same analysis on a set of data from Zhou et al. (2018) and Beylerian et al. (2020) obtained from the same participants as in the first part but where the sensitivity was measured using narrowband gratings (Figure 5). The contrast sensitivity measured using gratings and noise patches are presented in Figure 5A for controls and Figure 5B for amblyopes. In both groups, the sensitivity measured using gratings is higher and peaks at higher frequency, around 3 c/d (green curves in Figure 5A for controls; magenta curves in Figure 5B for amblyopes). The sensitivity of the amblyopic eye is also reduced with a lower peak frequency in both conditions (dashed curves, Figure 5B).

We then performed the same factor analysis as we had done in the first part. The eigenvalues for the 9 factors of each dataset are represented in form of scree plots in Figure 5C for control subjects and Figure 5D for amblyopic subjects. Accordingly, we again performed a varimax rotation of the first 2 components to generate the channel tuning curves. These factors tuning curves are reported in Figure 5E for controls and Figure 5F for amblyopes. The tuning curves from grating stimuli present a different tuning compared to the ones obtained with noise patches, peaking approximately one octave higher. In accordance with what was observed with noise patches, the tuning curves of both eyes of controls (green continuous and dashed lines) and of the FE of amblyopes (magenta continuous lines) are still very similar. However, a major difference is that the tuning curves of the AE (dark-magenta dashed lines) are tuned to lower frequency ranges compared to the FE (magenta continuous lines), resulting in a tuning that is quite similar to that from noise patches.

**Figure 6:**
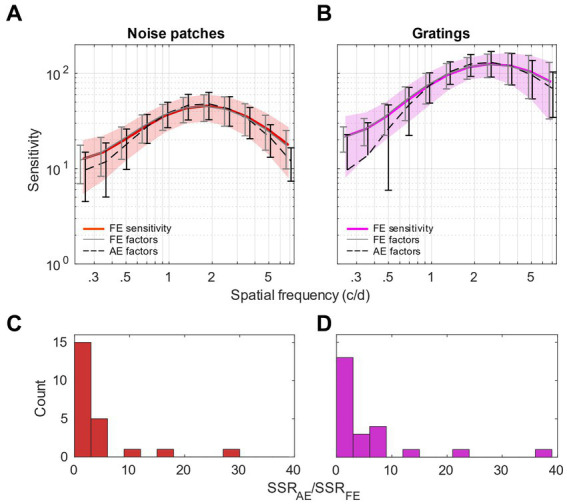
Confirmatory analysis. **(A,B)** Comparison of the sensitivity of the fellow eye to the sensitivity reconstructed from the factor loadings issued from the fellow eye (FE, continuous gray lines) or the amblyopic eye (AE, dashed black lines). **(A)** For noise patches. **(B)** For gratings. Shaded areas represent std. of the data. Error bars represent std. of the model fits across observers. Note that the bottom tails of some error bars are not represented as they were reaching negative values on a log-scale. **(C,D)** Histograms of the ratios of the Sum of Squared Residuals (SSR) between the model using the AE factor loadings and the FE factor loadings. **(C)** For noise patches sensitivity. **(D)** For gratings sensitivity.

To validate this observation, we performed a confirmatory analysis to compare the fits to the noise patches and gratings sensitivities in the fellow-eye by using factor loadings from the fellow or amblyopic eye, respectively. This analysis is presented in Figure 6. In Figure 6A, for the sensitivity to noise patches, we can observe, as expected, that the average sensitivity reconstructed from the FE loadings (gray continuous line) fits almost perfectly the raw sensitivity (red thick trace), and that the average sensitivity reconstructed from the AE loadings (black dashed line) fits very well the data too, although a bit less faithfully. In Figure 6B, for the sensitivity to noise gratings, we can make similar observations. However, it seems that the sensitivity reconstructed from the AE loadings (black dashed line) fits the data less accurately, particularly at low spatial frequency. This could be surprising as we observed that spatial frequency channels of FE were tuned to lower spatial frequencies for gratings. But actually, it can be explained by the fact that the least square regression will minimize the error in the range where the values are highest, i.e., for high spatial frequencies.

To quantify the regression quality using the factors from the AE and FE independently from the total variation and amplitude of the data, we calculated the ratios of the Sum of Squared Residuals (SSR) between the models using the AE factor loadings and the FE factor loadings. SSR measures the deviation unexplained by the regression model independently from the total variation. The histograms of the SSR ratios measured for noise patches and for gratings sensitivities are reported in Figures 6C,D, respectively. We can observe that the histogram for gratings sensitivity is slightly wider, with a mean SSR ratio of 5.70, larger than the ratio of 4.22 obtained with noise patches. More precisely, we performed a *F*-test between the SSR of the models using loadings of the AE and FE (vartest2 function from Matlab). The test revealed that the variances between the residuals are not statistically different for sensitivities to noise patches (*p* = 0.18), whereas they are significantly different for sensitivities to gratings (*p* < 0.001). This indicates that, for sensitivities to gratings, the loadings obtained from the AE are not able to faithfully describe the FE sensitivity; thereby confirming that the tunings between AE and FE are different for gratings but not noise patches.

## Discussion

We used multiple datasets containing different ranges of spatial frequency. However, despite the differences in the experimental designs and equipment, our exploratory factor analysis revealed very consistent spatial frequency channels across those studies. These spatial channels that govern the sensitivity at low and high spatial frequencies are remarkably similar between amblyopic and normal populations. For instance, their tunings have similar peaks and troughs as exemplified by the loadings. The channels that we observe here are also very similar to those in normal observers from the study of Peterzell and Teller (1996), who also applied the data reduction method to study the channels. In a second time, we found that the tunings of the spatial channels depend on how the contrast sensitivity is measured. To illustrate, if a grating rather than a noise pattern is used as the visual stimulus to obtain sensitivity of both normal and amblyopic observers, both low and high spatial frequency channels will have tunings that are shifted to a higher range of spatial frequencies than otherwise. This indicates that the tuning of the spatial channels depends on the presence of noise, which can shift the tuning to lower spatial frequencies (see Figure 5). However, this difference in the tuning of the spatial channels between the noise and grating was not observed from the amblyopic eye. Instead, when a grating patch was used to measure sensitivity, the amblyopic eye’s channels were atypically tuned to lower spatial frequencies and matched the channels obtained with noise patches. This shows that the presence of noise does not affect the tuning of the spatial channels in amblyopia at both low and high spatial frequencies.

Our surprising, initial observation that the tunings from the two spatial channels that were derived from sensitivity data using noise patches were identical in both eyes of controls and amblyopes led us presume that our results could have merely been an artifact from exploratory factor analysis. However, we obtained different tunings from the spatial channels obtained with gratings and noise patches, demonstrating that our results actually inform about the properties of spatial vision in amblyopia. Our second analysis also revealed that the amblyopic eye’s spatial channel (derived from grating data) has a unique tuning than those of FE, NDE, and DE. These observations show that factor analysis of contrast sensitivity data can lead to multiple outcomes and unique tuning functions from spatial channels, thereby indicating that our results were not constrained by the analysis procedure. In fact, it is worth noting that in this article, our main purpose was to compare the spatial frequency channels in controls and amblyopes. But some other analysis procedure or criterion could have led us to identify more numerous channels. Indeed, the selection of the number of components to pick in factor analysis is highly debatable and subject to interpretation (Jackson, 1993; Peres-Neto et al., 2005). In Table 2, we present the number of components suggested by 4 common methods that lead to inconsistent results. We opted for the most empirical way which was to look at the elbow of the scree plot and the number of factors that could describe more than 80% of the data’s variance (O’Rourke and Hatcher, 2013). Actually, it is interesting to notice that despite the higher variability observed in the sensitivity of the amblyopic eye (Figures 1B, 5B), the number of channels suggested by the different methods for the AE do not exceed the number of channels suggested for the FE. This shows that, despite large variability in the population, amblyopic contrast sensitivity is still governed by local correlations.

So, what could lead to such differences in the channels obtained using gratings or noise patches? The spatial frequency channels we report using noise patches are roughly equivalent to the ones reported by Peterzell and Teller (1996) using gratings. Gratings are much more narrowband than noise patches in both the spatial frequency and orientation domains. In our studies, this was even more marked at high spatial frequencies as the stimuli we used were of a fixed absolute size. Thus, it is possible that the narrow bandwidth of the high-spatial frequency gratings could have shifted the contrast sensitivity function and the spatial frequency channels toward higher spatial frequencies (Beylerian et al., 2020).

In fact, this could also explain why, with gratings, the spatial frequency channels obtained for the amblyopic eye were tuned to lower spatial frequencies, whereas there was no difference using noise patches. The broader bandwidth and external noise in the noise patterns could cover for the internal noise of the amblyopic eye (Levi and Klein, 2003; Levi et al., 2007), thus explaining why no difference was observed using noise patches. But the narrower bandwidth of the gratings would not cover for the internal noise of the amblyopic eye, thus resulting in a tuning shifted toward lower frequencies as observed with noise patches (which in fact would correspond to actual added external noise). So more generally, broader bandwidth and added noise, either external or internal would lead to lower spatial frequency tuning of the channels.

From the spatial frequency channels obtained using the contrast sensitivity measured with noise patches, we observed that the sensitivity in the low-SF channel could predict the sensitivity in the high-SF channel (Figure 4). However, despite a similar tuning, the high-frequency channel is much less represented in amblyopia (Figure 2). This finding concurs with the observation that the sensitivity of the 2 eyes of amblyopes is not correlated in either channel (Figure 3), whereas it is significantly correlated at low SF, and mildly correlated at high SF in controls (Figure 3). Altogether, this suggests that the sensitivities of the 2 eyes are similar in controls, likely resulting from a common brain processing (Barboni et al., 2020). Whereas they might be independent in amblyopia because of an altered processing of the amblyopic eye information by the brain. Such independent processes in amblyopia might also be supported by a lack of synchronicity between the 2 eyes (Chadnova et al., 2017; Reynaud and Hess, 2019; Wu et al., 2020; Gurman and Reynaud, 2022; Eisen-Enosh et al., 2023).

## Data availability statement

Publicly available datasets were analyzed in this study. This data can be found here: https://mvr.mcgill.ca/AlexR/data_en.html.

## Ethics statement

The studies involving humans were approved by the local ethics committees where the studies were conducted. The studies were conducted in accordance with the local legislation and institutional requirements. Written informed consent was obtained from all participants.

## Author contributions

AR and SM designed the study and wrote the manuscript. AR performed the analysis. All authors contributed to the article and approved the submitted version.
